# *W′* reconstitution modelling during intermittent exercise performed to task failure

**DOI:** 10.1007/s00421-025-05912-0

**Published:** 2025-08-11

**Authors:** Alexander J. Welburn, Charles F. Pugh, Stephen J. Bailey, Richard A. Ferguson

**Affiliations:** 1 School of Sport, Exercise and Health Sciences, Loughborough University, Loughborough, LE11 3TU UK; 2 School of Science and Technology, Nottingham Trent University, Nottingham, NG11 8NS UK

**Keywords:** Intermittent exercise, Cycling performance, Critical power, *W′* reconstitution *Wʹ*_BAL_

## Abstract

**Purpose:**

*W′* balance (*W′*_BAL_) modelling is becoming an important tool to monitor intermittent cycling performance. This study assessed the ability of different time constant (*τ*_*W*′_) equations for *W′* reconstitution (*W′*_rec_) to predict exhaustion during intermittent exercise and the relationship between parameters of *W′*_rec_ with established determinants of endurance performance.

**Methods:**

Thirteen cyclists performed cycling performance tests to determine: lactate threshold (LT), critical power (CP), *W′*, *V̇*O_2max_, maximal aerobic power (MAP) and maximal sprint power (*P*_max_). Participants subsequently performed three intermittent *Wʹ* depletion trials to volitional exhaustion involving different work and recovery periods: 20:10; 3 × 20 s intervals separated by 10 s recoveries before a final continuous effort, 60:30; 3 × 60 s intervals separated by 30 s recoveries before a final continuous effort, 20:10_TE_; repeated 20 s intervals each separated by 10 s recoveries. *W′*_BAL_ was determined via five different *τ*_*W*′_ equations and an individualised equation (*τ*_W′INDV_) calculated from the 20:10_TE_ under the assumption that the point of task failure represents 0 kJ.

**Results:**

Current *τ*_W′_ equations failed to predict exhaustion during intermittent exercise protocols to exhaustion. Total work done above CP for the 20:10_TE_ (*Wʹ*_total_20:10_TE_) was positively correlated with absolute and relative LT, CP, *V̇*O_2max_, MAP, and *P*_max_ (*r* = 0.64–0.80; *P* < 0.05). The *τ*_W′INDV_ was negatively correlated with relative CP (*r* = − 0.69), and LT_1_ (*r* = − 0.58), and *Wʹ*_total_20:10_TE_ (*r* = − 0.63).

**Conclusion:**

Individualised *τ*_W′_ should be utilised for the accurate prediction of *Wʹ*_BAL_. *W′*_rec_ is influenced primarily by aerobic performance parameters, including LT_1_ and CP.

## Introduction

The two parameters of the power-duration relationship during sustained severe-intensity exercise have become key performance markers within applied cycling performance science. The asymptote, critical power (CP), demarcates the heavy and severe exercise intensity domains (Jones et al. 2008; Poole et al. 1988, 2016), and is considered to reflect the greatest sustainable rate of oxidative metabolism in the absence of a progressive loss of muscle metabolic homeostasis. (Jones et al. 2010) The curvature constant, *W′*, represents a finite work capacity that can be continuously performed above CP (Jones and Vanhatalo 2017; Morton 2006; Poole et al. 1988). Both CP and *W′* can be assessed in the laboratory and field (Leo et al. 2022; Moritani et al. 1981; Poole et al. 1988; Spragg et al. 2022), which has allowed the CP model to become a versatile performance tool for athletes and coaches.

The CP model can reliably predict time to task failure during sustained severe-intensity exercise, however, it is unable to account for the recovery periods during intermittent exercise such that when power > CP, there is a proposed linear utilisation of *Wʹ* and when power < CP, there is a reconstitution of *Wʹ*. To address this, a model was developed to incorporate *W′* reconstitution *(W′*_rec_) using the CP model as parameter inputs (Skiba et al. 2012; Skiba & Clarke 2021). The balance between utilisation and reconstitution allows estimation of a *W′* value at a given time point during the intermittent exercise (*W′*_BAL_). Furthermore, as intermittent exercise continues, with inadequate recovery it is assumed exhaustion will occur or that the individual will no longer be able to perform work above CP when *Wʹ* eventually reaches 0 kJ. The ability to reconstitute *W′* is therefore key to performance observed through the ability to repeated high intensity efforts that can be crucial to overall cycling performance and training quality (Abbiss et al. 2013; Menaspà et al. 2017).

The early work of Skiba et al. (2012) developed an integral model, which allows the prediction of *W′*_rec_ using a curvilinear time constant, τ_W′_. This original time constant was derived from untrained individuals. Later work by Skiba et al. (2015) developed a differential model that uses a τ_W′_ derived from *W'* and the recovery work rate below CP (D_CP_). However, this variation is unable to be individualised in its current format, as only CP and *W′* are used. Subsequent work has indicated that *W′*_rec_ is faster in well-trained trained individuals (Bartram et al. 2018; Caen et al. 2021; Chorley et al. 2019). This may be attributable to differences in aerobic capacity, for example, individuals with higher *V̇*O_2peak_ and CP demonstrate faster rates of *W′*_rec_ (Bartram et al. 2018, 2022; Caen et al. 2021). There are now multiple *τ*_*W*′_ variations (Bartram et al. 2018, 2022; Pugh et al. 2022) that provide generalised models to allow *Wʹ*_BAL_ to be predicted in athletes. These are based on a power function with values fixed to the D_CP_, indicating that differences in CP between participants would lead to differences in *W′*_rec_. There appears to be a consensus that to further improve the accuracy of predicting *Wʹ*_BAL,_ *τ*_*W*′_ should be individualised (Bartram et al. 2022; Skiba et al. 2014), although there is limited insight on best practice.

While the physiological determinants of CP are well established (Goulding and Marwood 2023; Mitchell et al. 2018; Peden et al. 2024; Poole et al. 2016), the physiological determinants underpinning *Wʹ*, *W′*_rec_ and *Wʹ*_BAL_ remain somewhat elusive. *Wʹ* correlates with muscle volume in elite track cyclists (Kordi et al. 2021), and whilst no relationships have been observed with skeletal muscle fibre composition (Vanhatalo et al. 2016; Mitchell et al. 2018, Caswell et al. 2024), or capillarity (Mitchell et al. 2018), some facets of mitochondrial respiration may influence *Wʹ* (Peden et al. 2024). Nevertheless, it appears that depletion of *W′* is related to the reduction of metabolic substrates (phosphocreatine, PCr), and the accumulation of fatigue-related metabolites (H^+^, P_i_, ADP) to levels that can limit skeletal muscle function (Chidnok et al. 2013a, b, c; Ferguson et al. 2010; Fukuba et al. 2003; Poole et al. 1988). These contribute to the muscle fatigue process and subsequent task failure (Hostrup and Bangsbo 2017; Sundberg and Fitts 2019). Furthermore, the underpinning determinants of *W′*_rec_ have yet to be fully established with the main observations being correlations between parameters of *W′*_rec_ and CP (absolute, *r* = 0.52; relative, *r* = 0.57) and *V̇*O_2max_ (absolute, *r* = 0.8; relative, *r* = 0.62) (Caen et al. 2021; Chorley & Lamb 2020). Gaining a further insight into the association between established determinants of endurance performance with *W′*_rec_ and *τ*_*W*′_ will help guide further mechanistic exploration of the *Wʹ*_BAL_ model.

Research investigating *Wʹ*_BAL_ models have employed various CP testing and intermittent exercise protocols to determine generalised *τ*_*W*′_ functions, creating inconsistencies and making it difficult to compare model behaviour. This emphasises the need for a comprehensive understanding of how different *W′*_BAL_ models behave under the same circumstances and within the same population group, providing insight into where they converge or diverge, and what their inherent limitations are. Such understanding would establish clear boundaries for each model’s predictive capabilities and enable researchers to select appropriate *W′*_BAL_ models based on predetermined performance characteristics. This approach could inform the development of individualised testing protocols and guide future advances in *W′*_BAL_ modelling.

The aim of this study was two-fold: (1) assess different equations of *τ*_*W*′_ in the *W′*_BAL_ model to understand and identify which τ_W′_ value reflects a *W′*_BAL_ of 0 kJ at the point of exhaustion during three intermittent exercise protocols; (2) assess the relationship between cycling performance parameters with *W′*_rec_ parameters. It was hypothesised that; (1) there will be differences in *W′*_BAL_ at the point of task failure between the different *τ*_*W*′_ equations; (2) endurance performance parameters would correlate with the ability to complete more work during intermittent exercise and subsequently related to the ability to reconstitute *W′.*

## Methods

### Participants

Thirteen healthy individuals (10 males, 3 females, Table 1) volunteered to participate in the study. Participants were competitive cyclists training between 10 and 20 h per week, competing at the regional and national level. All completed health screening questionnaires prior to participation to mitigate for contraindications to maximal exercise. Participants did not have a history of cardiovascular, haematological, neuromuscular, or musculoskeletal abnormalities. Participants were fully informed of the risks and discomforts associated with all experimental trials before providing written, informed consent. All experimental procedures were approved by the Loughborough University Ethics Approvals Human Participants Sub-Committee (2021-6426-5519), and conformed to the Declaration of Helsinki, except for registration in a database.

**Table 1:** Participant characteristics and performance parameters

	Mean ± SD	Range
*Participant characteristics*		
Age (y)	24 ± 8	19–50
Height (m)	1.77 ± 0.07	1.64–1.86
Body mass (kg)	69.2 ± 6.6	57.2–81.0
*Performance Parameters*		
*V̇*O_2max_ (mL·min^−1^·kg^−1^)	58.2 ± 8.9	38.6–71.3
*V̇*O_2max_ (L·min^−1^)	4.0 ± 0.6	2.8–4.9
MAP (W)	371 ± 70	232–435
MAP (W·kg^−1^)	5.3 ± 0.8	3.7–6.7
CP (W)	269 ± 49	187–325
CP (W·kg^−1^)	3.9 ± 0.7	2.7–4.7
*W′* (kJ)	20.9 ± 6.3	8.6–28.6
*W′* (kJ·kg^−1^)	0.29 ± 0.08	0.14–0.40
LT_1_ (W)	193 ± 37	130–240
LT_1_ (W·kg^−1^)	2.8 ± 0.5	1.9–3.5
LT_2_ (W)	256 ± 42	185–305
LT_2_ (W·kg^−1^)	3.7 ± 0.6	2.7–4.4
*P*_max_ (W)	1215 ± 307	627–1723
*P*_max_ (W·kg^−1^)	17.5 ± 4.4	9.1–24.9
[Bla^−^]_peak_ mmol·L^−1^	14.7 ± 3.1	6.7–18.3
[BLa^−^]_min_ mmol·L^−1^	2.1 ± 0.8	0.5–3.3
[BLa^−^]_clr_ mmol·L^−1^ min^−1^	0.48 ± 0.10	0.24–0.59

[Bla^−^]_peak_ & [Bla^−^]_min_ highest and lowest value obtained 3 and 12 min testing; [BLa^−^]_clr_ (peak–min)/time between those two values from 3 and 12 testing; CP, critical power; LT_1_ first lactate threshold (baseline + 0.4 mmol·L^−1^); LT_2,_ second lactate threshold (fixed blood lactate concentration of 4 mmol·L^−1^); MAP, maximal aerobic power; *P*_max,_ maximal sprint power; *V̇*O_2max,_ maximal oxygen uptake; *W′*, curvature constant

### Experimental protocol

Participants attended the laboratory on seven occasions over a four-week period for the determination of; lactate thresholds, *V*^*˙*^O_2max_ and maximal aerobic power (MAP), CP and *W′* using 3- and 12 min fixed duration time-trial (TT) efforts, assessment of peak lactate [Bla^−^]_peak_ and lactate clearance rate [BLa^−^]_clr_ after the 3 min fixed duration TT, maximal sprint power (P_max_), and three intermittent *Wʹ* depletion trials performed to volitional exhaustion. Each visit was separated by at least 48 h.

All exercise tests were performed on the participant’s own racing bicycle attached to a commercially available cycle trainer (Kickr, Wahoo Fitness, Georgia, USA), which was calibrated prior to each use according to the manufactures guidelines. Power data was recorded at 1 Hz and downloaded into WKO5™ (Training Peaks™, Louisville, USA) and processed in Microsoft Excel. All performance trials were conducted at the participants freely chosen pedal cadence. Prior to each performance trial (except the lactate threshold, and *V*^*˙*^O_2max_ tests), participants performed a standardised warm up involving 5 min at 50 W and 100 W, 3 min at 55% and 65% of MAP, before a final 5 min at 50 W. Participants were instructed to maintain a normal diet during the testing period and refrain from consuming alcohol and caffeine during the 24 h preceding testing. All tests were conducted in constant laboratory ambient conditions (19–21 °C, 40–50% humidity).

### Test procedures

Lactate thresholds, *V̇*O_2max_ and MAP.

Participants performed a step incremental test to determine the first (LT_1_) and second (LT_2_) lactate thresholds. The test commenced at 100 W for males and 70 W for females with power output being increased by 30 W every 4 min until 90% of age predicted max heart rate (220 beats.min^−1^–age) was achieved. Capillary blood samples (20 µL) were collected from the fingertip at rest (baseline) and during the final 30 s of each stage and analysed for lactate concentration within three hours (Biosen C-line analyser, EKF Diagnostics, UK). A lactate-power curve was produced for each participant and fitted with a 3rd order polynomial, with lactate threshold parameters defined as: LT_1_, baseline + 0.4 mMol L^−1^ (Bourdon 2013) and LT_2_, fixed lactate concentration of 4 mMol·L^–1^ (Kindermann et al. 1979).

After 30 min of rest, participants completed an incremental step test to determine *V̇*O_2max_ and MAP. Following a warm-up for 5 min at 50 W, the test began at 150 W for males and 100 W for females for 1 min, after which power increased 25 W every 60 s, in a step-wise manner, until volitional exhaustion or when pedal cadence fell 10% below the chosen cadence for ~ 5 s. Breath-by-breath pulmonary gas exchange was measured continuously throughout exercise (Vyntus-CPX; CareFusion, Hoechberg, Germany). The system had been calibrated with known O_2_ and CO_2_ concentrations and a 3 L volume syringe. *V̇*O_2max_ and MAP were defined as the highest *V̇*O_2_ and power output for a 30 s and 60 s period during the test, respectively.

CP and *W′* were estimated using two fixed-duration TT efforts to achieve the highest average power possible (Simpson and Kordi 2017, Coakley and Passfield 2018). Participants completed two rolling-start TT for 3 and 12 min, where gear ratio and cadence were self-selected. Participants were instructed and encouraged to complete each TT with the greatest average power output possible and to ensure they were completely exhausted at the end of each effort. This method was performed twice over three separate visits. On one visit, participants performed both the 3- and 12-min test protocols (same-day procedure), and on the other two visits the 3- and 12-min tests were performed on separate days (separate-day procedure).

Same-day procedure: Following the standardised warm-up, participants performed the 3-min TT. Immediately after the effort participants recovered passively for approximately 1–2 min, before cycling at 50 W for 30 min (resulting in a total recovery period of 32 min). Capillary blood samples (20 uL) were collected at 1, 2, 3, 4, 6, 8, 10, 15, 20, 25, 30 min following the TT. After the final blood sample was obtained and the 12-min test commenced. Blood samples were analysed for blood lactate concentration and peak lactate ([Bla^−^]_peak_) and lowest lactate ([Bla^−^]_min_) determined as the highest and lowest concentrations recorded, respectively, and lactate clearance rate [BLa^−^]_clr_ was calculated as ([BLa^−^]_peak_—[BLa^−^]_min_/duration from peak to lowest value). Separate day procedure: Participants attended the laboratory on two occasions separated by 48 h. On each of the two occasions, after the warm-up was completed, participants first performed a 6 s maximal sprint to determine *P*_max_ (peak 1 s power). Participants chose their own resistance via gear choice with the ergometer in resistance mode, with the sprint starting from rolling start (< 40 rev·min^−1^). Participants recovered by cycling for 20 min at 50 W before performing either the 3- or 12-min TT as described above. The highest value from either of the two 6 s maximal sprints was used for *P*_max_.

The parameters of the power-duration relationship, CP and *Wʹ*, were calculated using the linear inverse-time model (Eq. 1)

1 $$P = W^{\prime} \cdot \frac{1}{T} + {\mathrm{CP}}$$

where *P* is the given power output above CP, and t is the time to task failure (s) and *W′* is the total work (*J*) completed above CP.

Intermittent *W*′ depletion trials.

Three intermittent trials were completed, on separate occasions, in a randomised order. Each trial consisted of different work and recovery periods performed until volitional exhaustion or when pedal cadence fell 10% below the chosen cadence for ~ 5 s. Power output for the work periods (*P*_work_) was determined using Eq. 2.

2 $$\begin{gathered} P_{work} = \, P_{6} + \, 0.5 \, * \, \Delta {\mathrm{CP}} \hfill \\ P_{6} = \, \left( {W^\prime /360s} \right) \, + {\text{ CP}} \hfill \\ \end{gathered}$$

where *P*_6_ is the time to exhaustion in 6 min, and ΔCP represents the difference between CP and *P*_6_. The highest CP and *W*′ values obtained through the same day and separate CP assessments were selected for each participant and used to calculate *P*_work_. Specifically, in two participants values were used from the separate day trials and in 11 participants values were used from the same day procedure. The recovery power output was set at 50% of LT_1_ (P_rec_).

Intermittent *W′* depletion trial 1 (20:10_TE_). This consisted of repeated 20 s intervals at P_work_, each separated by 10 s recovery periods at *P*_rec_ and performed continuously until exhaustion.

Intermittent *W′* depletion trial 2 (20:10). This consisted of 3 × 20 s intervals at *P*_work_, each separated by 10 s recovery periods at *P*_rec_ before a final continuous effort at *P*_work_.

Intermittent *W′* depletion trial 3 (60:30). This consisted of 3 × 60 s intervals at *P*_work_, each separated by 30 s recovery periods at *P*_rec_ before a final continuous effort at *P*_work_. During these trials, participants were permitted to view duration, power, and cadence during the initial 3 efforts of the 60:30 and 20:10, however, during the final exhaustive effort (and throughout the 20:10_TE_) no feedback other than cadence was provided.

Power data from the three intermittent exercise trials, along with CP and *W′* were used to calculate *W′*_BAL_ using the ordinary differential equation (ODE) (Pugh et al. 2022; Skiba and Clarke 2021) (Eq. 3).

3 $$W^{\prime}_{{{\mathrm{BAL}}}} = \left\{ {\begin{array}{*{20}c} {W^{\prime}_{{{\text{bal, }}i - 1}} - \left( {\left[ {Pi - {\mathrm{CP}}} \right]\cdot \Delta \mu_{i} } \right), Pi > {\mathrm{CP}}} \\ {W^{\prime}_{0} - W^{\prime}_{{{\mathrm{expended}}}} \cdot \left( {e^{{ - }{\frac{{\Delta \mu_{i} }}{\tau W^{\prime}}}} } \right),Pi < {\mathrm{CP}}} \\ \end{array} } \right.$$

where *i* = the *i*^th^ segment of the total time subdivided into n segments at 1 Hz, ($\Delta \mu )$, ${P}_{i}$ = mean power output for the segment *i*. *W*′_BAL_ is calculated sequentially and ${W^{\prime}}_{\mathrm{BAL}}{,}_{i -1}$ represents the preceding estimation of *W′*_BAL_. *W′*_expended_ is the quantity of the depleted *W′* at $i -1$ and is calculated (Eq. 4).

4 $$W^{\prime} _{{{\mathrm{expended}}}} = W^{\prime} _{0 } - W^{\prime} _{{{\text{bal, }}i - 1}}$$

The reconstitution time constant (*τ*_*W*′_) for the *W′*_BAL_ was calculated via five different equations.

The first used Eq. 5 (Skiba et al. 2012), an exponential regression obtained from the integral model, referred to as Skiba_1_.

5 $${\mathrm{Skiba}}_{1} \left[ {\tau_{{W^{\prime}}} } \right] = 546{\mathrm{e}}^{{\left( { - 0.01DCP} \right)}} + 316$$

The second used Eq. 6 (Skiba et al. 2015) from the differential model, which is scaled against the size of *W′* to *D*_CP_, referred to as Skiba_2_.

6 $${\mathrm{Skiba}}_{2} [\tau_W^{\prime} ] = W\prime /D_{{{\mathrm{CP}}}}$$

The next were calculated using the power function of *D*_CP_—$\tau w^{\prime}$ (Eq. 7)

7 $$\tau_{W\prime } = A \cdot D_{{{\mathrm{CP}}}}^{ - B}$$

where *A* represents the scaling factor, and *B* represents the rate of decay.

The third used Eq. 8 (Bartram et al. 2018).

8 $${\mathrm{Bart}}\left[ {\tau_{W\prime } } \right] = 2287.2 \cdot D_{{{\mathrm{CP}}}}^{ - 0.688}$$

The fourth and fifth used Eqs. 9 and 10 (Pugh et al. 2022), respectively.

9 $${\mathrm{Nat}}\left[ {\tau_{W\prime } } \right] = 1883 \cdot D_{{{\mathrm{CP}}}}^{ - 0.487}$$

10 $${\mathrm{Reg}}[\tau_{W\prime } ] = 5184 \cdot D_{{{\mathrm{CP}}}}^{ - 0.700}$$

An individualised *τ*_*W*′_ (*τ*_*W*′INDV_) was calculated from the 20:10_TE_ where a single *τ*_*W*′_ was calculated for each participant with the average D_CP_ of across all recovery periods under the assumption that at the point of task failure represents a *W′*_BAL_ of 0 kJ. Time-to-exhaustion (TTE) and total work done above CP for the 20:10_TE_ (*W′*_total_20:10_TE_) were calculated for the 20:10_TE_ trial.

### Statistical analysis

Data were normally distributed as assessed by Shapiro–Wilk’s test (*P* ≤ 0.05). An initial 2-way ANOVA was performed to assess differences in *W′*_BAL_ with trial and *τ*_*W*′_ as the independent variables. One-way ANOVA were subsequently used to assess differences in *W′*_BAL_ between the five *τ*_*W*′_ equations for each trial. Where significant effects were observed, Bonferroni-corrected post-hoc *t-tests* were used to locate differences. When Mauchly’s test of sphericity indicated that the assumptions of sphericity had been violated, Greenhouse–Geisser correction was used. Relationships between *W′*, *W′*_total_20:10_TE_, and *τ*_*W*′INDV_ with physiological and performance measures were analysed using Pearson′s product-moment correlation coefficient. Significance was accepted at *P* ≤ 0.05. Data are presented as mean ± standard deviation (SD), unless otherwise stated.

## Results

Participant characteristics and performance parameters are shown in Table 1.

### ***W′***_BAL_ modelling

**Fig. 1:**
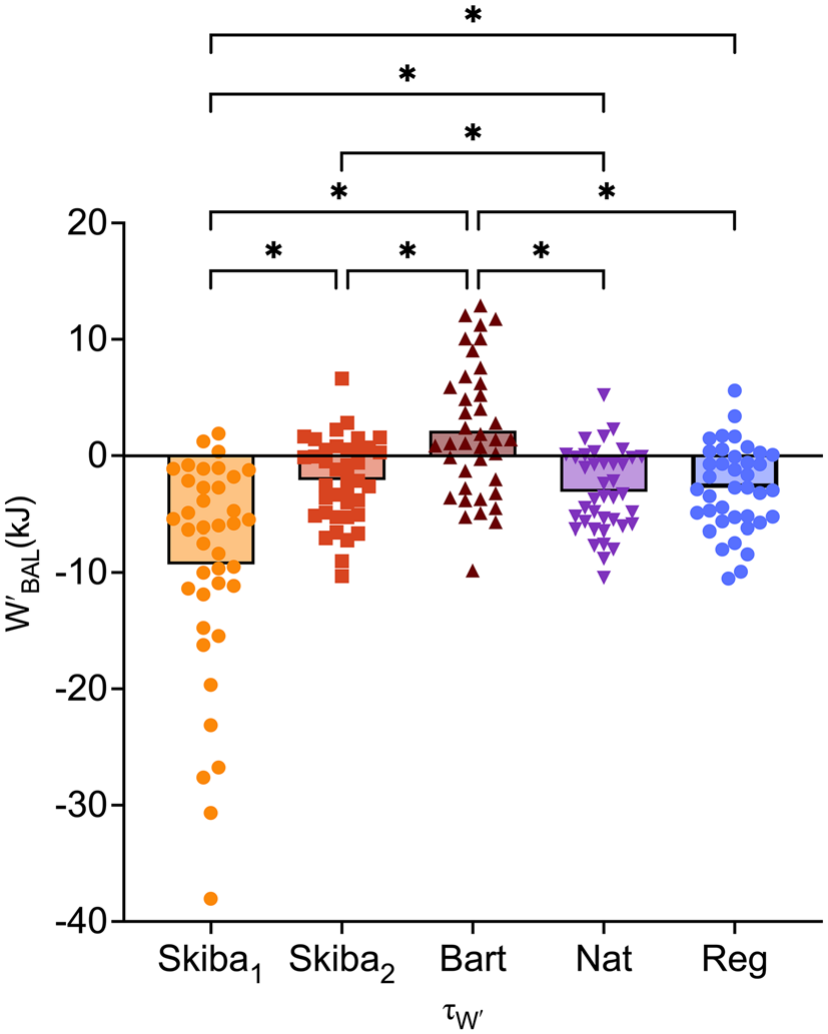
*W′*_BAL_ at the point of task failure for all intermittent depletion trials calculated using each *τ*_*W*′_ model. Mean and individual data are shown. * P ≤ *0.05*

*Wʹ*_BAL_ at the point of task failure for each intermittent depletion trial, using each τ_W′_ modelling method are shown in Fig. 1. There was no main effect for trial (*F*_(2,24)_ = 1.121, *P* = 0.342, *η*_*p*_^2^ = 0.085), however, there was a main effect for *τ*_*W*′_ (*F*_(1.507,18.080_ = 64.956, *P* < 0.001, *η*_*p*_^2^ = 0.844) and an interaction between trial x *τ*_*W*′_ (*F*_(1.419,17.032)_ = 42.497, *P* < 0.001, *η*_*p*_^2^ = 0.780).

When combining the three intermittent trials for analysis, there were significant differences (*P* < 0.001) between: Skiba_1_[*τ*_*W*′_] and Skiba_2_[*τ*_*W*′_] (7.2 ± 1.4 kJ); Skiba_1_[*τ*_*W*′_] and Bart[*τ*_*W*′_] (11.5 ± 2.1 kJ); Skiba_1_[*τ*_*W*′_] and Nat[*τ*_*W*′_] (6.2 ± 1.3 kJ); Skiba_1_[*τ*_*W*′_] and Reg[*τ*_*W*′_] (6.5 ± 0.4); Skiba_2_[*τ*_*W*′_] and Bart[*τ*_*W*′_] (4.3 ± 0.8 kJ); Skiba_2_[*τ*_*W*′_] and Nat[*τ*_*W*′_] (− 1.0 ± 0.3 kJ); Bart[*τ*_*W*′_] and Nat[*τ*_*W*′_] (− 5.3 ± 0.9 kJ); Bart[*τ*_*W*′_] and Reg[*τ*_*W*′_] (− 4.9 ± 0.8 kJ).

**Fig. 2:**
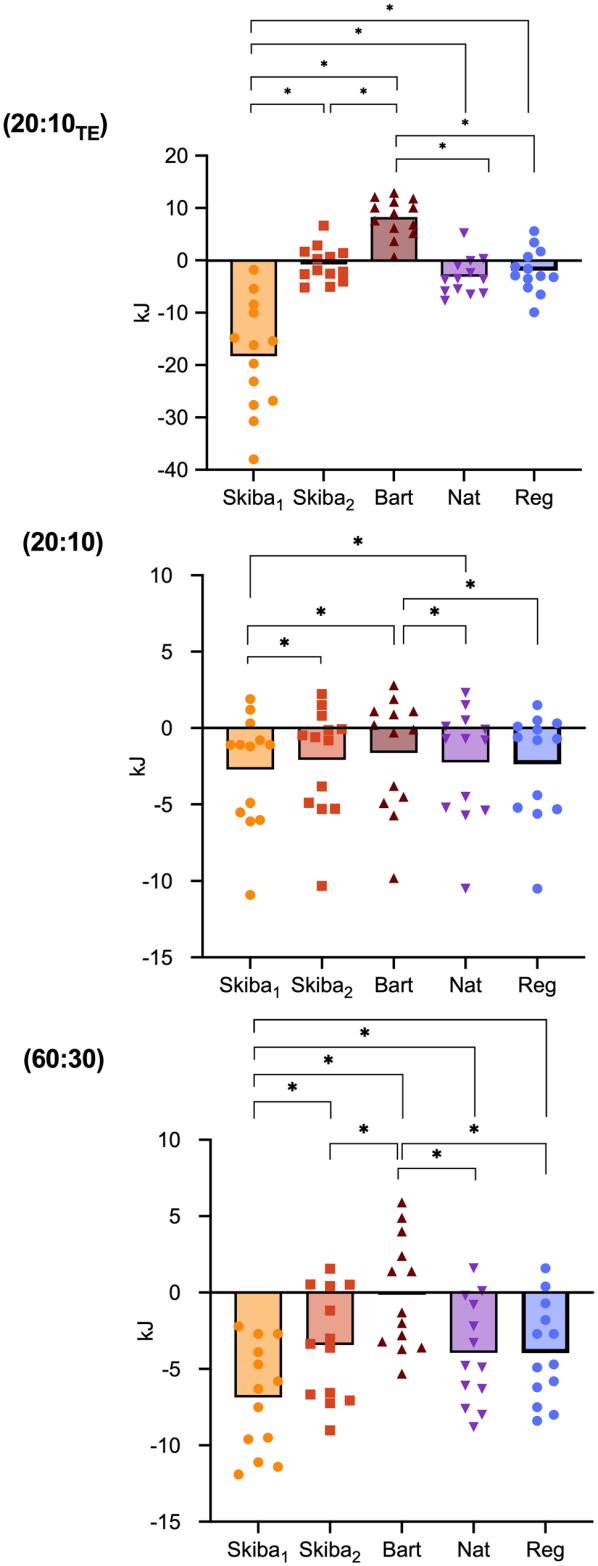
*W′*_BAL_ at the point of task failure for each intermittent depletion trial (20:10 _TE_; 60:30; 20:10), calculated using each *τ*_*W*′_. Mean and individual data are shown. *P ≤ *0.05*

During the 20:10_TE_ trial (Fig. 2), *W′*_BAL_ calculated using Skiba_1_[τ_W′_] was lower compared to Skiba_2_[*τ*_*W*′_] (− 17.4 ± 3.4 kJ, *P* < 0.001); Bart[τ_W′_] (− 26.6 ± 3.4 kJ, *P* < 0.001); Nat[*τ*_*W*′_] (− 15.1 ± 2.2, *P* < 0.001) and Reg[τ_W′_] (− 16.3 ± 2.5, *P* < 0.001). When calculated using Skiba_2_[*τ*_*W*′_], *W′*_BAL_ was lower compared to Bart[*τ*_*W*′_] (− 9.0 ± 1.5 kJ, *P* < 0.001). When calculated using Bart[*τ*_*W*′_], *W′*_BAL_ was higher compared to Nat[*τ*_*W*′_] (11.4 ± 1.3 kJ, *P* < 0.001) and Reg[*τ*_*W*′_] (10.2 ± 1.2 kJ, *P* < 0.001). There was no difference between Nat[τ_W_] and Reg[τ_W′_].

During the 20:10 trial (Fig. 2), *W′*_BAL_ calculated using Skiba_1_[τ_W′_] was lower compared to Skiba_2_[*τ*_*W*′_] (− 0.6 ± 0.1 kJ, *P* < 0.001); Bart[*τ*_*W*′_] (− 1.1 ± 0.2 kJ, *P* < 0.001) and Nat[*τ*_*W*_] (− 0.5 ± 0.1 kJ, *P* < 0.001). When calculated using Bart[τ_W′_], *W′*_BAL_ was higher compared to Nat[*τ*_*W*_] (0.6 ± 0.01 kJ, *P* < 0.001) and Reg[*τ*_*W*_] (0.7 ± 0.2 kJ, *P* = 0.007); There was no difference between Nat[*τ*_*W*_] and Reg[*τ*_*W*′_].

During the 60:30 trial (Fig. 2), *W′*_BAL_ calculated using Skiba_1_ [τ_W′_] was lower compared to Skiba_2_[*τ*_*W*′_] (− 3.4 ± 0.2 kJ, *P* < 0.001); Bart[τ_W′_] (− 16.7 ± 0.6 kJ, *P* < 0.001), Nat[*τ*_*W*′_] (− 2.9 ± 0.3 kJ, *p* < 0.001) and Reg[*τ*_*W*′_] (− 2.9 ± 0.4 kJ, *p* < 0.001). When calculated using Skiba_2_ [*τ*_*W*′_], *W′*_BAL_ was lower compared to Bart[*τ*_*W*′_] (− 3.3 ± 0.5 kJ, *P* < 0.001). When calculated using Bart[*τ*_*W*′_], *W′*_BAL_ was higher compared to Nat[*τ*_*W*′_] (3.8 ± 0.4 kJ, *P* < 0.001) and Reg[*τ*_*W*′_] (3.8 ± 0.4 kJ, *P* < 0.001). There was no difference between Nat[*τ*_*W*′_] and Reg[*τ*_*W*′_].

### Individualised ***τ***_W′_

**Fig. 3:**
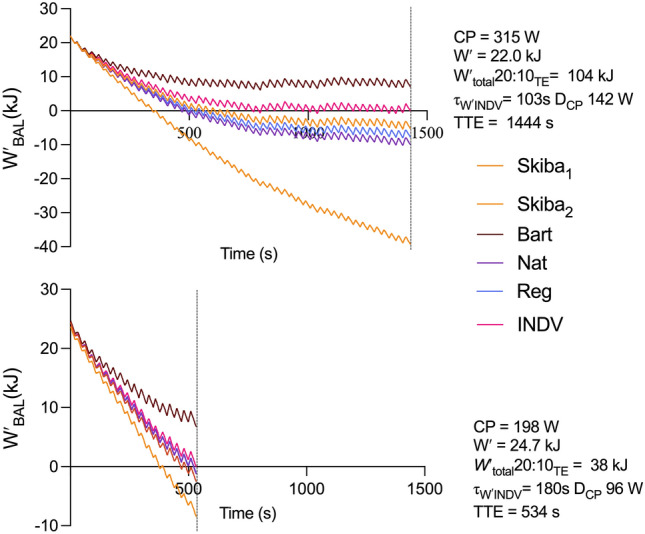
*W′*_BAL_ in two participants with different CP characteristics throughout the 20:10_TE_ trial calculated using the five *τ*_*W*′_ models and τ_W′INDV._ Vertical line indicates task failure

*τ*_*W*′INDV_ were calculated from the 20:10_TE_ trials under the assumption that the point of task failure represents a *W′*_BAL_ of 0 kJ. Figure 3 demonstrates, in two participants with contrasting CP values, real time *W′*_BAL_ modelling during the 20:10_TE_ trials using the five *τ*_*W*′_ models as well as the τ_W′INDV_. τ_W′INDV_ calculated for all participants is shown in Table 2.

**Table 2:** Individual performance values, τ_W′INDV_ and average D_CP_ during intermittent depletion trial 20:10_TE_

ID	CP	*W′*	τ_W′INDV_ (s)	*D*_CP_ (W)	TTE	*W′*_total_20:10_TE_	*W′*_total_20:10_TE_/*W′*
	(W)	(kJ)			(s)	(kJ)	(%)
1	306	21.1	***	183	768	49.5	235
2	264	22.6	134	123	740	30.3	134
3	315	22.1	103	142	1444	103.5	468
4	272	28.1	189	154	769	47.6	169
5	298	24.5	115	121	1070	56.0	229
6	317	24.0	115	134	1127	86.5	360
7	325	25.4	149	148	978	58.9	232
8	195	13.7	132	79	1603	41.6	304
9	268	28.6	124	95	697	63.4	222
10	245	12.0	134	127	1274	42.0	350
11	187	8.6	168	123	678	17.3	201
12	301	16.1	108	143	1100	60.8	378
13	198	24.7	180	96	534	38.2	155
Mean	269	20.9	138	128	983	53.5	264
SD	49	6.3	28	28	323	22.7	100

CP, critical power; *D*_CP_, recovery work rate below CP; TTE, time to exhaustion during 20:10_TE_; τ*W′*_INDV_, individualised time constant of *Wʹ*_rec_; *W′*, curvature constant; *W′*_total_20:10_TE_, total work done completed above critical power during 20:10_TE_ until task failure; *W′*_total_20:10_TE_/*W′*, percentage ratio between *W′*_total_20:10_TE_ and *W′*. Note*** = unable to calculate individual τ_W′_

**Fig. 4:**
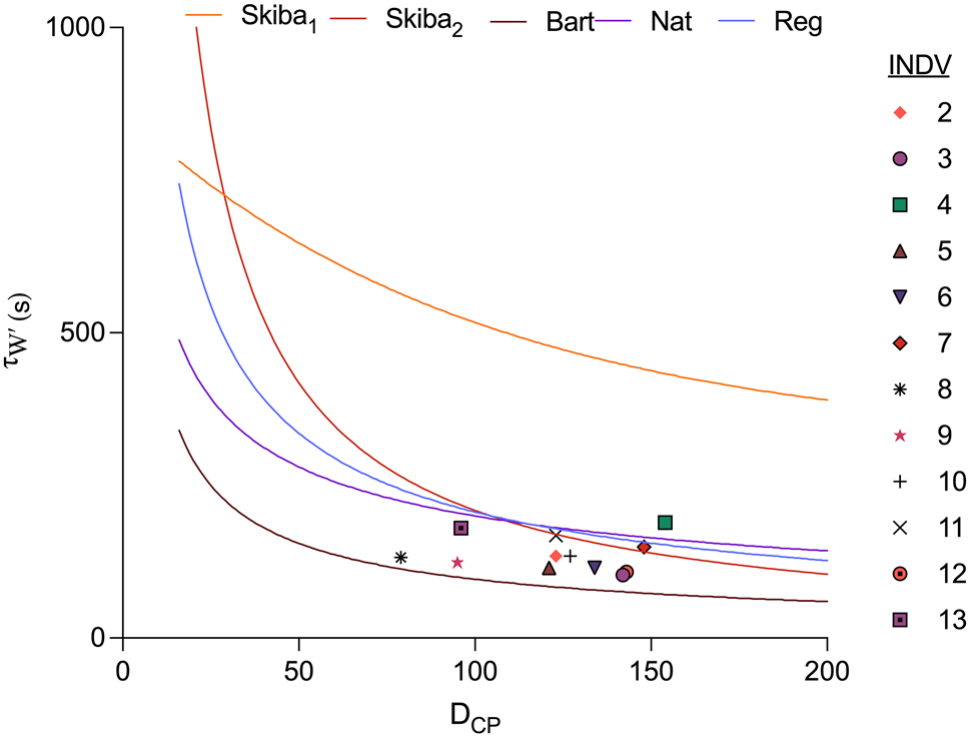
τ_W′INDV_ obtained from the 20:10_TE_ trial plotted in relation to D_CP_. Also plotted are the relationships between *τ*_*W*′_ and *D*_CP_ for the five *τ*_*W*′_ equations; Skiba_1_[*τ*_*W*′_], Skiba_2_[*τ*_*W*′_] fitted as mean *W*′ (20.8 kJ)/*D*_CP_, Bart[*τ*_*W*′_], Nat[*τ*_*W*′_] and Reg[*τ*_*W*′_] (see Methods). *Note* unable to calculate individual *τ*_*W*′_ for participant 1

In Fig. 4, individual *τ*_*W*′_ values obtained from the 20:10_TE_ trial in Table 2 are plotted in relation to D_CP_. Also plotted are the relationships between *τ*_*W*′_ and D_CP_ for the five *τ*_*W*′_ equations; Skiba_1_[*τ*_*W*′_], Skiba_2_[*τ*_*W*′_], Bart[*τ*_*W*′_], Nat[*τ*_*W*′_] and Reg[*τ*_*W*′_] (see Methods). At the D_CP_ utilised in the present study (i.e., 50% of LT_1_), the *τ*_*W*′INDV_ values calculated for each participant fell between Bart[*τ*_*W*′_], Nat[*τ*_*W*′_] and Reg[*τ*_*W*′_] except participant 4.

### ***W′***_BAL_ correlates

**Fig. 5:**
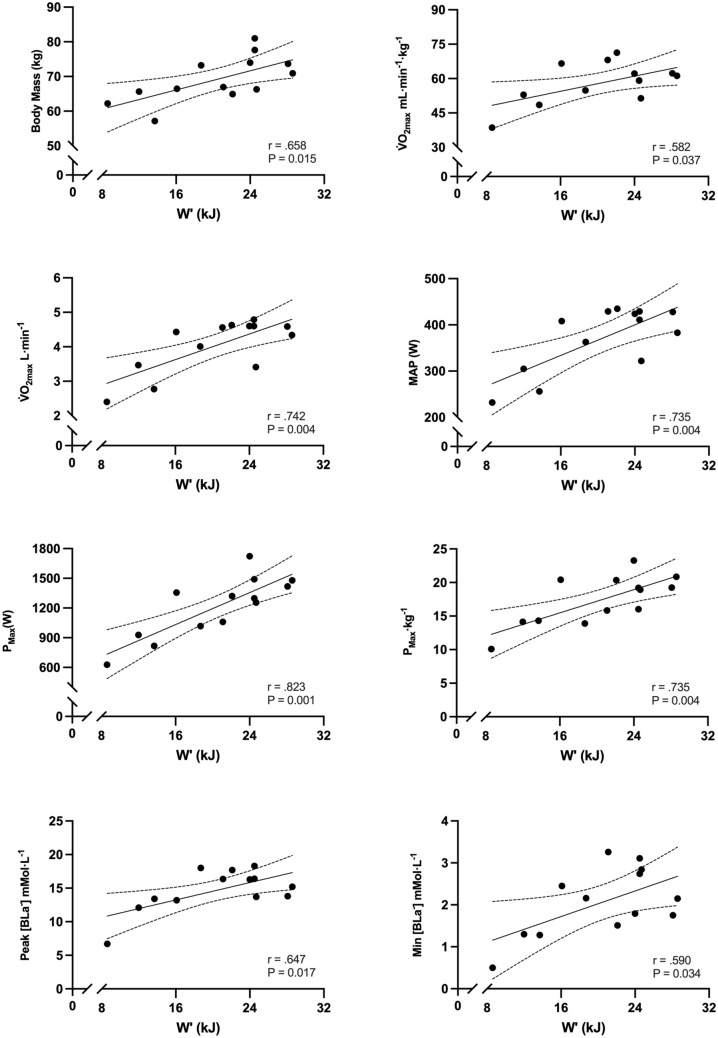
Correlations between *W′* and physiological and performance parameters (95% CI are also shown)

**Fig. 6:**
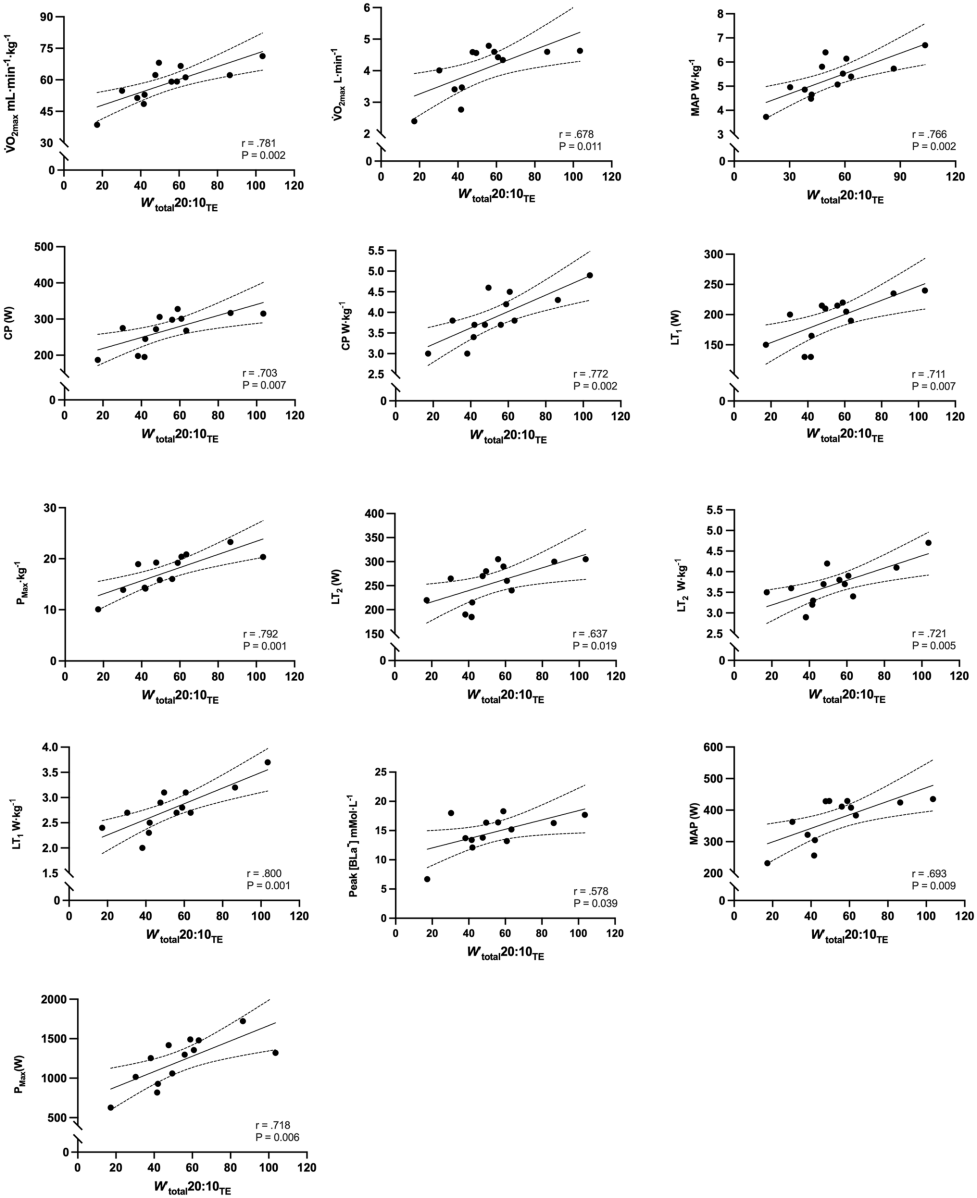
Correlations between *Wʹ*_total_20:10_TE_ and physiological and performance parameters (95% CI are also shown)

**Fig. 7:**
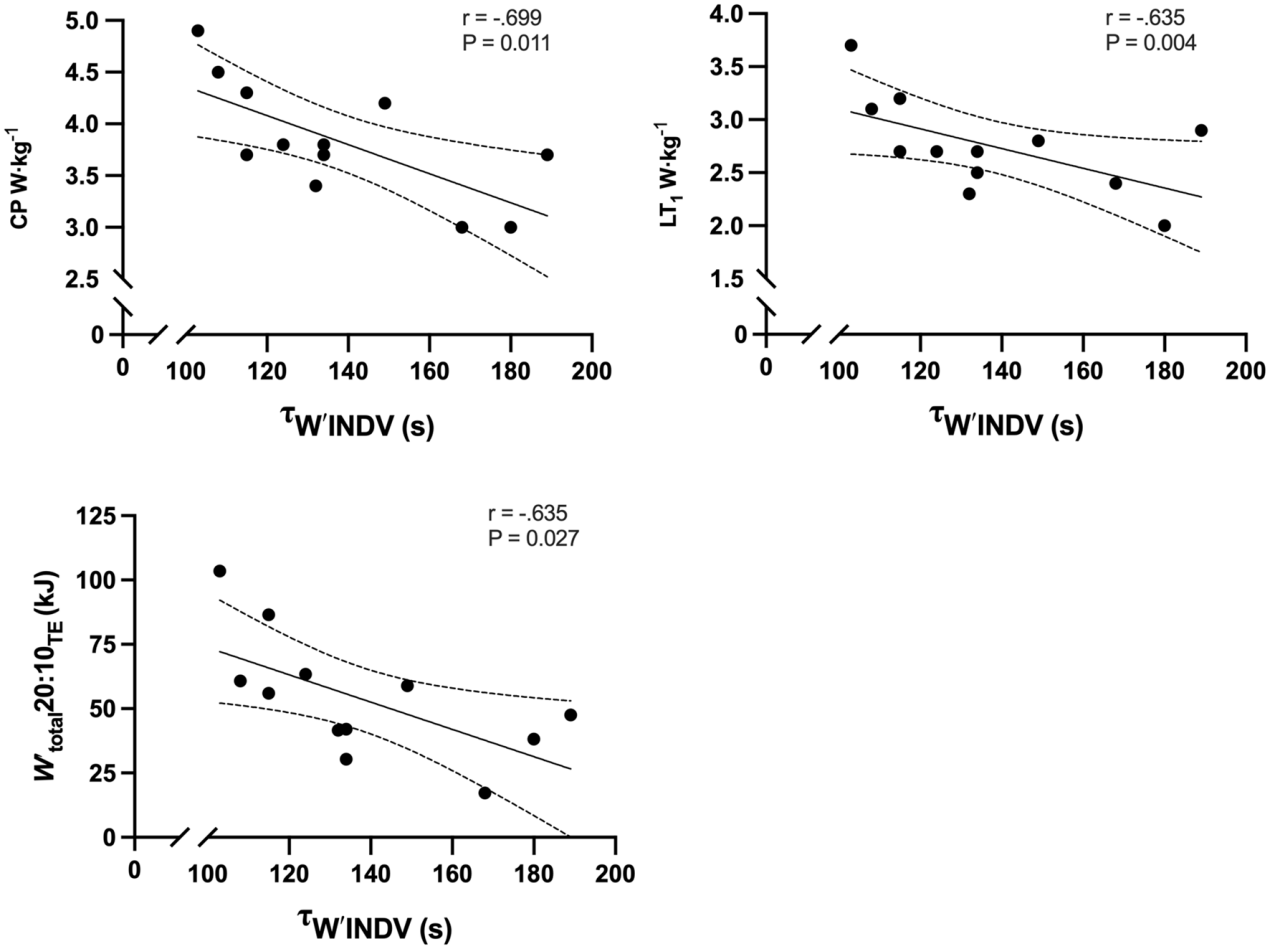
Correlations between τ_W′INDV_ and physiological and performance parameters (95% CI are also shown). *Note* unable to calculate individual *τ*_*W*′_ for participant 1, therefore, *n* = 12

Correlations of *W′*, *W′*_total_20:10_TE_, and τ_W′INDV_ with physiological and performance parameters are shown in Table 3. *W′* was positively correlated with body mass, absolute and relative *V̇*O_2max_, absolute MAP, absolute and relative *P*_max_, [BLa^−^]_peak,_ and [BLa^−^]_min_ (Fig. 5). *W′*_total_20:10_TE_ was positively correlated with absolute and relative *V̇*O_2max_, absolute and relative MAP, absolute and relative CP, absolute and relative LT_1_, absolute and relative LT_2_, absolute and relative P_max_, and [BLa^−^]_peak_ (Fig. 6). The τ_W′INDV_ was negatively correlated with relative CP, relative LT_1_, and *W′*_total_20:10_TE_ (Fig. 7).

**Table 3:** Correlations between *W′*, *W′*_total_20:10_TE_, and τ_W′INDV_ and physiological and performance parameters

	*W′* (kJ)	*W′*_total_20:10_TE_ (kJ)	*τ*_*W*′INDV_ (s)
Body mass (kg)	r = 0.658 P = 0.015 *	r = 0.216 P = 0.478	r = − 0.053 P = 0.870
V̇O_2max_ (mL·min^−1^·kg^−1^)	r = 0.582 P = 0.037 *	r = 0.781 P = 0.002 *	r = − 0.525 P = 0.080
V̇O_2max_ (L·min^−1^)	r = 0.742 P = 0.004 *	r = 0.678 P = 0.011 *	r = − 0.393 P = 0.207
MAP (W)	r = 0.735 P = 0.004 *	r = 0.693 P = 0.009 *	r = − 0.435 P = 0.157
MAP (W·kg^−1^)	r = 0.543 P = 0.055	r = 0.766 P = 0.002 *	r = − 0.333 P = 0.291
CP (W)	r = 0.504 P = 0.879	r = 0.703 P = 0.007 *	r = − 0.543 P = 0.068
CP (W·kg^−1^)	r = 0.252 P = 0.407	r = 0.772 P = 0.002 *	r = − 0.699 P = 0.011 *
*W′* (kJ)	– –	r = 0.478 P = 0.09	r = − 0.060 P = 0.852
*W′* (W′·kJ^−1^)	– –	r = 0.497 P = 0.084	r = 0.077 P = 0.812
LT_1_ (W)	r = 0.506 P = 0.078	r = 0.711 P = 0.007 *	r = − 0.457 P = 0.135
LT_1_ (W·kg^−1^)	r = 0.266 P = 0.078	r = 0.800 P = 0.001 *	r = − 0.588 P = 0.044 *
LT_2_ (W)	r = 0.474 P = 0.102	r = 0.637 P = 0.019 *	r = − 0.441 P = 0.151
LT_2_ (W·kg^−1^)	r = 0.474 P = 0.554	r = 0.721 P = 0.005 *	r = − 0.575 P = 0.151
*P*_max_ (W)	r = 0.823 P = 0.001 *	r = 0.718 P = 0.006 *	r = − 0.237 P = 0.459
*P*_max_ (W·kg^−1^)	r = 0.735 P = 0.004 *	r** = **0.792 P = 0.001 *	r = − 0.286 P = 0.368
[BLa^−^]_peak_ mmol·L^−1^	r = 0.647 P = 0.017 *	r** = **0.578 P = 0.039 *	r = − 0.424 P = 0.170
[BLa^−^]_min_ mmol·L^−1^	r = 0.590 P = 0.034 *	r = 0.140 P = 0.647	r = − 0.115 P = 0.722
[BLa^−^]_clr_ mmol·L^−^	r = 0.427 P = 0.146	r = 0.514 P = 0.056	r = − 0.438 P = 0.154
Endurance Ratio	r = *0.805* P = 0.056	r = 0.050 P = 0.872	r = 0.404 P = 0.193
*W*_total_20:10_TE_ (kJ)	r = 0.478 P = 0.090	– –	r = − 0.635 P = 0.027 *

[Bla^−^]_peak_ & [Bla^−^]_min_ highest and lowest value obtained 3 and 12 min testing; [BLa^−^]_clr_ (peak–min)/time; CP, Critical power; Endurance ratio, curvature constant/critical power; LT_1_ first inflection point calculated as baseline + 0.4 mmol·L^−1^; LT_2_, Fixed blood lactate concentration of 4 mmol·L^−1^; MAP, maximal aerobic power; P_max,_ maximal sprint power; V̇O_2max,_ maximal oxygen uptake; *W'*, curvature constant; *W′*_total_20:10_TE_, work done above critical power during 20:10_TE_ until task failure. *P ≤ 0.05

## Discussion

The main findings of this study are as follows: (1) current τ_W′_ equations used to calculate *W′*_rec_ failed to predict exhaustion during intermittent exercise protocols to exhaustion; (2) when utilising the *Wʹ*_BAL_ model *τ*_*W*′_ should be individualised for accurate prediction; (3) multiple physiological performance characteristics including LT_1,_ LT_2_, CP, *V̇*O_2max_, MAP, *P*_max_, and [BLa]_peak_ were positively correlated with *Wʹ* and *Wʹ*_total_20:10_TE_; (4) relative CP, LT_1_, and *Wʹ*_total_20:10_TE_ were negatively correlated with τ_W′INDV_ (Fig. 7).

According to the *Wʹ*_BAL_ model, it is assumed that when *Wʹ* is depleted, i.e., 0 kJ, exhaustion will occur, or the individual will no longer be able to perform work above CP (Chorley & Lamb 2020; Jones et al. 2010; Skiba et al. 2012). However, the five equations of τ_W′_ used to model *Wʹ*_BAL_ failed to predict a value of 0 kJ at the point of exhaustion during the intermittent *Wʹ* depletion trials. The original time constant described by Skiba et al., (2012); Skiba_1_[τ_W′_] clearly underpredicted the *Wʹ*_BAL_ at the point of exhaustion, particularly in the 20:10_TE_ and 60:30 trials, with no values above 0 kJ being reported. There was an improvement in the prediction when using Skiba_2_[τ_W′_], which had the lowest mean value during the 20:10_TE_ trial (Fig. 2). Bart[*τ*_*W*′_], however, often overpredicted *Wʹ*_BAL_, particularly in the 20:10_TE_. The time constants described by Pugh et al., (2022) resulted in slight improvements in prediction compared to Skiba_1_[τ_W′_] and Bart[τ_W′_], particularly in the 20:10_TE_ and provided values much closer to 0 kJ.

The reasons for the poor predictive capability of current *τ*_*W*′_ equations are complex and multifaceted. It is likely that the curvilinear responses of *W′*_rec_ through the mono-exponential *τ*_*W*′_ function during the recovery phase of exercise are either too fast or slow such that *Wʹ*_BAL_ at the point of exhaustion is either over or under predicted. This is likely to be a function of the complex processes of skeletal muscle energetics, including PCr utilisation and recovery (Chidnok et al. 2013a, b, c; Skiba et al. 2015) and O_2_ delivery and utilisation (Korzeniewski & Rossiter 2020; Lievens et al. 2024) interacting with the key intermittent exercise protocol parameters including interval work rate and duration, recovery power (normalised recovery power relative to CP; *D*_CP_) and duration (Caen et al. 2019; Skiba et al. 2014). For example, whilst one of our protocols utilised a 60:30 work:recovery ratio, similar to that used by (Skiba et al. 2014), the D_CP_ values were substantially different e.g., with D_CP_ based on a 20 W recovery power compared to 50% LT_1_ (resulting in a D_CP_ of 129 W). τ_W’_ equations from Pugh et al. (2022) were derived from D_CP_’s of 25, 50 and 100 W with work:recovery ratio of 30:60. Furthermore, individual participant performance capacities (e.g., LT_1_, CP, *V̇*O_2max_, *P*_max_), and physiology (e.g., muscle fibre composition) are also likely to interact. For example, the average CP in our population was 269 W compared to 240 W for recreational athletes in Skiba et al. (2012), ~ 380 W for competitive track cyclists in Pugh et al. (2022) and 393 W for elite team pursuit cyclists in Bartram et al. (2018). Consequently, the generalised *τ*_*W*′_ values used were unable to fully capture the intricacies in these protocol and participant performance capacities to accurately predict 0 kJ at the point of exhaustion.

To achieve more accurate predictions, additional parameters would be required to input into the *Wʹ*_BAL_ model. This might include individual participant performance and physiological characteristics that have been shown to correlate with *W′*_rec_ parameters. Whilst not a *W′*_rec_ parameter per se, *Wʹ*_total_20:10_TE_ reflects how much *W′* was used during intermittent exercise, indicating a great ability to reconstitute *W′* and was positively correlated to *P*_max_, *V*^*˙*^O_2max_, MAP, CP, LT_1_, LT_2_ and [BLa^−^]_peak_. In addition, the individualised *τ*_*W*′_ values were negatively correlated with LT_1_ and CP (when expressed relative to body mass). Given the close relationships between these performance parameters and skeletal muscle morphological and metabolic properties (Mitchell et al. 2018; Peden et al. 2024), it might be suggested that skeletal muscle characteristics such as muscle fibre type distribution, capillarity, and mitochondrial content/function play an important role and permit a faster rate of rate of *W′*_rec_. Furthermore, *W′* was positively correlated to body mass, *P*_max_, *V*^*˙*^O_2max_, MAP, and [BLa-]_peak_ supporting previous observations that *W′* is highly dependent on muscle strength and size parameters (Kordi et al. 2018; Kordi et al. 2021)_._

The inherent limitations of the 2-parameter model at work rates above the severe domain likely cause *W′*_BAL_ models to underestimate *W′* depletion rates, potentially affecting calculated *W′*_rec_ values. Our observation of positive relationships between performance parameters, including *P*_max_, suggests that incorporating a 3-parameter CP model may enhance *W′*_BAL_ prediction accuracy by improving *W′* precision in the extreme domain, though this is beyond the scope of the current study. Regardless, adding components to *Wʹ*_BAL_ models increases complexity, and the mathematisation of physiology remains challenging, particularly given uncertainty about how these components would influence *W′*_rec_.

Whilst our focus has been on *W′*_rec_ during the recovery period, the *Wʹ*_BAL_ model works on the assumption that *Wʹ* utilisation is linear when work is being performed above CP, which is built on the assumption (Morton 2006) that mechanical efficiency remains constant during exercise. This is particularly important since it is established that mechanical efficiency is not constant over time, particularly during intense dynamic exercise (González-Alonso et al. 2000), and repeated intense exercise (Bangsbo et al. 2001; Krustrup et al. 2001, 2003). In fact, mechanical efficiency declines as exercise progresses, which is dependent upon ATP production from the different metabolic pathways involved throughout exercise i.e., PCr hydrolysis, glycolysis, and oxidative phosphorylation. Additionally, there are suggestions that *Wʹ* is not a fixed work capacity (Chidnok et al. 2013c; Dekerle et al. 2015) rather it may be more of a dynamic work capacity which is influenced by work rate and total *Wʹ* depletion (Welburn et al. 2024). Collectively, these observations highlight the complexity of modelling *Wʹ* utilisation and reconstitution, and further investigation is warranted to explore the constraints and boundaries of the *W′*_BAL_ model.

The present study was not without limitations. Accurate and reliable determination of CP and *W′* are essential for *W′*_BAL_ modelling. Large errors in the measurement of these parameters will reduce the confidence in an accurate *W′*_BAL_ prediction. For example, a larger error with *W′* will have implications for the prediction of time to failure. CP also influences the D_CP_ value, which directly impacts the mathematical function to predict *W′*_BAL_ at any given point. In the present study, CP and *W′* were determined using two fixed-duration TT efforts (Simpson and Kordi 2017). Whilst this method provides a valid and reliable assessment of CP and *W′*, it is not possible to determine the level of measurement error. However, these highlight the balance between the applicability of *W′*_BAL_ modelling in the field for elite athletes and more accurate laboratory-based assessment. Finally, more detailed modelling of *W′*_rec_ has been suggested by Caen et al (2021), who reported a bi-exponential recovery pattern with separate τ_*W′*_ values for fast and slow phases of reconstitution. However, the intermittent protocols in the present study adopted 10 s and 30 s recovery periods, thus the slow phase was unlikely to be captured.

In conclusion, this study suggests the Reg[*τ*_*W′*_] or Skiba_2_[*τ*_*W*′_] equations could be used in generalised *Wʹ*_BAL_ modelling during intermittent exercise with short recovery periods. However, the framework for Skiba_2_[*τ*_*W*′_] does not allow for individualisation in its current format, therefore, an individualised *τ*_*W*′_ should be utilised for a more accurate prediction of *Wʹ*_BAL_. There were correlations between parameters of *W′*_rec_ and multiple physiological performance characteristics, including LT_1_ and CP, suggesting that *W′*_rec_ is influenced primarily by aerobic parameters, which could be incorporated into further *Wʹ*_BAL_ models. From a practical perspective, we suggest that the specific needs of the athlete should be considered, and individualised intermittent exercise protocols utilised in which intensity and duration-specific parameters are adopted. Such considerations could enhance the use of *Wʹ*_BAL_ modelling as a powerful performance metric, allowing the quantification of ‘recoverability’.

## Data Availability

Data are available upon reasonable request.

## Abbreviations

[BLa^−^]_clr_: Rate of blood lactate clearance between the 3- and 12-min effort
[Bla^−^]_min_: Lowest blood lactate concentration obtained after the 3-min time trial effort
[Bla^−^]_peak_: Highest blood lactate concentration obtained after the 3-min time trial effort
ADP: Adenosine diphosphate
CP: Critical power
*D*_CP_: Recovery work rate below CP
H^+^: Hydrogen ion
LT_1_: First lactate threshold, defined as baseline + 0.4 mmol L^−^^1^
LT_2_: Second lactate threshold, defined as fixed blood lactate concentration of 4 mmol L^−^^1^
MAP: Maximal aerobic power
MMP: Mean maximal power
ODE: Ordinary differential equation
PCr: Phosphocreatine
*P*_i_: Inorganic phosphate
*P*_max_: Peak 1 s power
*P*_work_: Work rate intensity during interment exercise
TTE: Time to exhaustion
*V*^*˙*^O_2max_: Maximal oxygen uptake
*Wʹ*: Curvature constant
*Wʹ*_BAL_: Balance between *Wʹ* depletion and *Wʹ* reconstitution during intermittent exercise
*Wʹ*_rec_: *Wʹ* Reconstitution
*Wʹ*_total_20:10_TE_: Total work done completed above critical power during 20:10_TE_ until task failure
τ_W__′_: Time constant to recover 63% of *Wʹ*
*τ*_W__′INDV_: Individualised time constant of *Wʹ* reconstitution
